# Plant Physiology is recruiting Assistant Features Editors for 2024

**DOI:** 10.1093/plphys/kiad283

**Published:** 2023-05-13

**Authors:** Yunde Zhao, Mary Williams

**Affiliations:** Editor-in-Chief, Plant Physiology; Section of Cell and Developmental Biology, University California San Diego, 9500 Gilman Drive, La Jolla, California 92093-0116, USA; Features Editor, American Society of Plant Biologists

The mission of the Assistant Features Editor (AFE) program is to help disseminate exciting discoveries published in *Plant Physiology* and to advance young scientists' careers. This past January, *Plant Physiology* welcomed 19 new AFEs to the editorial board. Together with AFEs recruited in 2022, these young scientists have brought their passion for science to the journal, communicating to our readers some of the most exciting *Plant Physiology* papers through News and Views articles. Some examples of News and Views written by AFEs can be found at https://academic.oup.com/plphys/issue/192/1.

Our Assistant Features Editor program greatly advances young scientists' careers; in fact, of those who have completed the program and moved into their next roles, the majority have secured tenure track positions. *Plant Physiology* Assistant Features Editors receive professional advice on scientific writing from our seasoned editors, and the Assistant Features Editors have abundant opportunities to network with our regular editors. For example, Assistant Features Editors conduct informal interviews with *Plant Physiology* editorial board members, enabling the Assistant Features Editors and *Plant Physiology* readers to get to know our editors (see https://plantae.org/research/plant-physiology/#editor-profiles). Assistant Features Editors are invited to the *Plant Physiology* editorial board meetings, with their travel expenses covered by the journal. At the editorial board meeting, the Assistant Features Editors not only can interact with and learn from senior scientists but also contribute to discussions on journal policies and operation. One of our AFEs recently shared, “I really enjoy writing N&V and can already see improvement in my writing style!” and another wrote, “The editorial meeting last year was great. It was very interesting to learn about topics of concern for this journal and hear the discussions to try and mitigate them. The AFE program is amazing and I am very grateful to be part of it.”

We are now recruiting a new cohort of AFEs to join the *Plant Physiology* editorial board, replacing some of the current members who will step down from their roles with the journal at the end of 2023. This new cohort will work with the journal for 24 months beginning in January 2024. The new cohort will be invited to attend the *Plant Physiology* Editorial board meeting and the centennial celebration of ASPB in June 2024 in Hawaii.

If you are interested in becoming an AFE, we are welcoming applications through Monday, October 2, 2023. Further details of the activities of the AFEs and how to apply are available online at https://plantae.org/plant-physiology-is-recruiting-assistant-features-editors-for-2024/.

We are interested in hearing about your current position and future goals and why you are interested in the position. We will need your Curriculum Vitae and the contact details of two individuals familiar with your work as a researcher. We will also expect two samples of your writing, one a first-authored paper and the other a sample News and Views commentary you write on your selection of one of the *Plant Physiology* articles listed at https://plantae.org/plant-physiology-is-recruiting-assistant-features-editors-for-2024/. We hope to have selected the new AFEs in late November.

